# The association between emotional abuse in childhood and healthcare utilization in adulthood among sami and non-sami: the SAMINOR 2 questionnaire survey

**DOI:** 10.1186/s12913-024-11211-9

**Published:** 2024-06-21

**Authors:** Farhiyo A. Osman, Astrid M.A. Eriksen, Anja M. Davis Norbye

**Affiliations:** 1 Department of Community Medicine, UiT the Arctic University of Norway, Hansine Hansens veg 18, Tromsø, 9019 Norway; 2 Centre for Sami Health Research, Department of Community Medicine, UiT the Arctic University of Norway, Hansine Hansens veg 18, Tromsø, 9019 Norway; 3 Department of Health and Care Sciences, UiT the Arctic University of Norway, Hansine Hansens veg 18, Tromsø, 9019 Norway

**Keywords:** Emotional abuse in childhood, Adverse childhood experience, Somatic and mental specialist healthcare, SAMINOR 2, Indigenous, Cross-sectional study

## Abstract

**Background:**

Emotional abuse in childhood is the most common type of childhood abuse worldwide and is associated with a variety of somatic and mental health issues. However, globally and in indigenous contexts, research on the associations between emotional abuse in childhood and somatic and mental specialist healthcare utilization in adulthood is sparse.

**Aim:**

The main aim of this study was to investigate the association between emotional abuse in childhood and somatic and mental specialist healthcare utilization in adulthood in Sami and non-Sami populations, and to examine whether this association differs between the two ethnic groups.

**Method:**

This study used cross-sectional data from the SAMINOR 2 Questionnaire Survey - a population-based study on health and living conditions in areas with Sami and non-Sami populations in Middle and Northern Norway. In total, 11 600 individuals participated in SAMINOR 2. Logistic regression was used to present the association between emotional abuse in childhood and somatic and mental specialist healthcare utilization.

**Results:**

Emotional abuse in childhood was significantly associated with somatic specialist healthcare utilization in adulthood (fully adjusted odds ratio [OR] 1.31, 95% confidence interval [CI] 1.15–1.49), with no differences observed between ethnic groups. Emotional abuse in childhood was also associated with mental specialist healthcare utilization (fully adjusted OR 3.99, 95% CI 3.09–5.14), however this association was weaker among Sami (crude OR 2.38, 95% CI 1.37–4.13) compared with non-Sami (crude OR 5.40, 95% Cl 4.07–7.15) participants.

**Conclusions:**

Emotional abuse in childhood is associated with somatic and mental specialist healthcare utilization in adulthood, with a stronger association to mental healthcare utilization. The association between emotional abuse in childhood and mental specialist healthcare utilization was weaker among Sami than non-Sami participants. Future studies should investigate the reason for this ethnic difference. Our results highlight the need to strengthen efforts to prevent childhood abuse and develop strategies to reduce its societal and personal burden.

## Introduction

According to the World Health Organization [1], around 1 billion (approximately half of) children aged 2–17 years have been impacted by various forms of childhood abuse, including physical, sexual, and emotional abuse [2]. Prior studies have primarily focused on merged categories of violence and abuse or on sexual and physical abuse alone, with less attention to the specific impacts of emotional abuse [3, 4]. The limited existing research has demonstrated an association between emotional abuse in childhood and somatic and mental health outcomes [3, 5–8]. The wide definition of emotional abuse in childhood includes isolated incidents, as well as patterns of non-verbal, hostile behaviours or attitudes toward children, which can cause them to experience fear, guilt, powerlessness, humiliation, or a sense of not being wanted [8]. Emotional abuse in childhood is highly prevalent [5, 9]. According to a meta-analysis by Stoltenborgh et al. [9], the prevalence was 36% for emotional abuse, 22.6% for physical abuse and 12.7% for sexual abuse during childhood, respectively.

The adverse impacts that emotional abuse in childhood have on health equal those that result from physical and sexual abuse [8, 10]. A comprehensive review and meta-analysis [8] examined the long-term consequences of physical and emotional abuse in childhood, on both somatic and mental health outcomes. The results revealed that emotional abuse was strongly associated with a range of psychological disorders, including symptoms of depression, anxiety disorders, and post-traumatic stress disorder (PTSD). Emotional abuse showed the strongest connection to these mental health conditions, while the association with somatic health was more inconsistent [8]. A Norwegian study suggested a strong association between abuse in childhood, including emotional abuse, and adult mental health issues [11]. Although several risk factors for emotional abuse exist [1], the direction of association remains somewhat unclear. Some studies report equal risk of psychological maltreatment between boys and girls [12], however a Norwegian report from 2014 describe a higher prevalence among women regarding psychological abuse from parents or guardians in childhood [13]. Regarding overall violence, there seem to be declining prevalence in younger age cohorts, indicating that older age cohorts are associated with higher risk of reporting childhood violence [13]. Low socioeconomic status in families has been associated with an increased risk of emotional abuse in childhood [14], as well as being a minority [15], although the direct correlation is not clear.

Although the long-term negative impacts of emotional violence in childhood on health in adulthood have been acknowledged, little is known about the long-term impacts of emotional violence in childhood on healthcare utilization globally or in an indigenous context. Research on childhood abuse in indigenous populations is based on limited data. However, some research has indicated that childhood violence is more common among indigenous than non-indigenous populations [16–19]. This has been linked to the cultural experience the indigenous populations have had to endure, such as harsh assimilation policies, colonization, and discrimination [16, 20, 21]. A study exploring healthcare utilization for specific diseases, like arthritis, suggested that indigenous people from Canada, New Zealand, and the United States utilize specialist healthcare less than non-indigenous people, but they are more likely to be hospitalized than their non-indigenous counterparts [22]. A Canadian study showed that indigenous people utilize primary and specialist healthcare less frequently, but they are hospitalized more frequently than the non-indigenous population [23]. The Sami are the indigenous inhabitants of Norway, Sweden, Finland, and Russia’s Kola Peninsula. In Norway, the Sami live primarily in the northern region, where they constitute the majority in some areas and the minority in others. The culture, language, and traditions of the Sami are distinct from those of the majority population. A population-based study from Norway showed that the Sami population reported a higher prevalence of emotional abuse in childhood than non-Sami from the same geographical area [16]. Emotional abuse was found to be strongly associated with chronic pain and mental health problems in adulthood [6, 7].

The few studies that have examined healthcare utilization among indigenous people from Norway indicated a similar level of healthcare utilization between Sami and non-Sami. Gaski and colleagues [24] compared specialist healthcare spending in Sami-majority and Sami-minority in neighbouring municipalities and found no significant differences, whereas another study examining healthcare use among the youth population found equal frequency of GP and mental healthcare use, but Sami youth having a higher odds for using school healthcare service [25]. One population-based master thesis suggested that primary healthcare utilization was similar between Sami and non-Sami adults [26].

### Knowledge gap

There is a lack of research examining the associations between exposure to abuse in childhood and somatic and mental specialist healthcare utilization in adulthood among Sami and non-Sami, and this is especially true regarding outcomes related to emotional abuse in childhood. To our knowledge, this is the first population-based study examining the association between emotional abuse in childhood and somatic and mental specialist healthcare utilization in adulthood in an area with Sami and non-Sami populations in Norway. Our aim was to investigate the association between emotional abuse in childhood and somatic and mental specialist healthcare utilization in adulthood, and to examine whether this association differed between Sami and non-Sami populations.

## Materials and methods

### The SAMINOR study

**Fig. 1:**
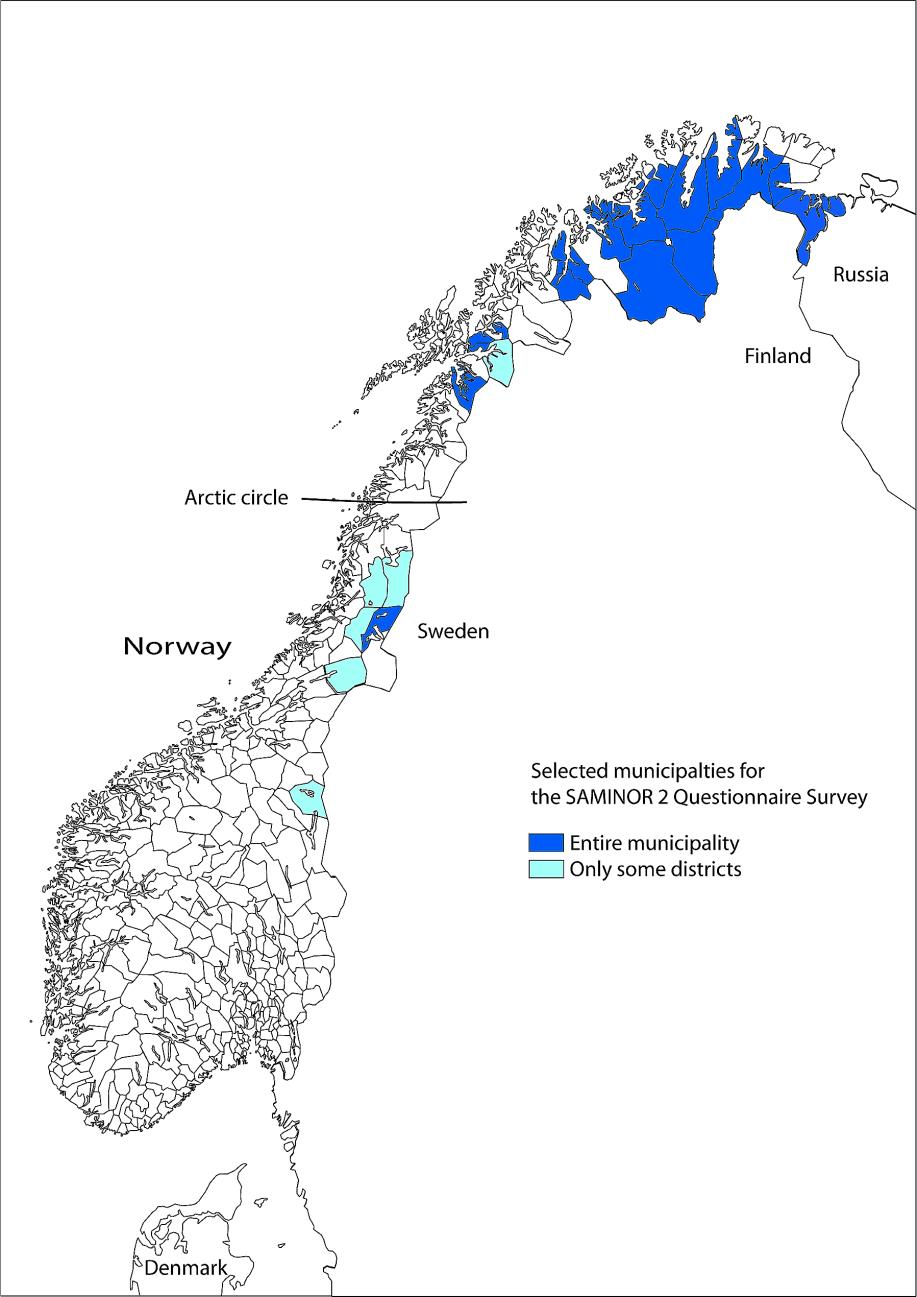
Study area for SAMINOR 2 questionnaire study The map of the study area is used with permission from the centre for Sami Health Research (CSHR) at UiT the Arctic University of Norway. It is designed by Marita Melhus at CSHR, based on a raw map of Norway from the Norwegian Mapping Authority.

The SAMINOR study is the first population-based study on health and living conditions in areas with Sami and non-Sami populations in Norway [27, 28]. It consists of two surveys; SAMINOR 1 (2003–2004) and the SAMINOR 2 Questionnaire Survey (2012), in addition to a SAMINOR 2 clinical examination (2014). This study used the SAMINOR 2 Questionnaire Survey (hereafter SAMINOR 2). The details of SAMINOR 2 are available elsewhere [27]. Participants from 25 municipalities or smaller districts within municipalities in Central and Northern Norway were invited to SAMINOR 2 (Fig. 1), selected based on the distribution of the Sami population [27].

### Study sample

The present study used a cross-sectional design and included questionnaire data from SAMINOR 2, in which 44 669 men and women aged 18–69 years were invited to participate. In total, 11 600 individuals completed the questionnaire and consented to participate (27% response rate). Observations with missing values were excluded.

### Variables

#### Exposure variable

The exposure variable was emotional abuse in childhood. The SAMINOR 2 questionnaire included the NorVold Abuse Questionnaire (NorAQ), a validated questionnaire to measure childhood abuse [29, 30]. One question in the NorAQ was used to measure emotional abuse in childhood: “Have you experienced that someone systematically and over time has tried to repress or humiliate you?” Participants who responded “yes, as a child” were classified as “having experienced emotional abuse in childhood”, while participants who responded “no” were categorized as “not having experienced emotional abuse in childhood”. Participants who responded, “yes, as a child and adult” and “yes, as an adult” were excluded. We have no information on who carried out the violence.

#### Outcome variables

Outcome variables were somatic and mental specialist healthcare utilization. Somatic specialist healthcare utilization in adulthood was measured by the following question: “In the last 12 months, have you been examined or treated for physical problems at any of the following?” Response options were: “hospital”, “specialist medical centre”, “private practice specialist”, and “none of the above”. Somatic specialist healthcare utilization among participants who responded “none of the above” was classified as non-use; all other responses were classified as “somatic specialist healthcare utilization in adulthood”.

Mental specialist healthcare utilization was measured by the following question: “In the last 12 months, have you been examined or treated for psychological problems at any of the following?” Response options were: “hospital”, “specialist medical centre”, “private practice specialist”, and “none of the above”. Mental specialist healthcare utilization among participants who responded “none of the above” was classified as “non-use”; all other responses were classified as “mental specialist healthcare utilization in adulthood”.

#### Possible interaction term

As differences in the prevalence of emotional abuse in childhood have been found between Sami and non-Sami in Norway [16], and as the two ethnic groups have different cultures, languages, and religions that may affect the associations under investigation, we decided to examine whether ethnicity could be an interaction term. The question, “What ethnicity do you consider yourself?” was used to determine ethnicity. Participants who responded “Sami” were categorized as Sami, while participants who responded “Norwegian” were categorized as non-Sami. Participants who responded “Kven” or “other” were excluded from the analyses. Thus, the variable ethnicity had two values: “Sami” and “non-Sami”.

#### Possible confounders

Age and gender: Data on age and gender were collected from the national registry Statistics Norway (SSB). Due to a low number of young adult participants in SAMINOR 2, we merged the age groups 18–29 and 30–39 to ensure anonymity, which was a requirement in the original master thesis. Age is presented in categories as 18–39, 40–49, or 50–69 years. Gender is presented as male and female.

Educational level: Educational level was included as a proxy for socioeconomic status. Educational level was determined from the question: “How many years of schooling have you completed?”, which is a frequently used measure of education [31]. For the dataset on somatic specialist healthcare utilization, the responses were categorized into primary school (≤ 9 years), upper secondary school (10–12 years), higher university or college (14–15 years), and university (≥ 16 years). Due to the small number of participants, in the dataset on mental specialist healthcare utilization, educational level was categorized as 0–12 years and ≥ 13 years to ensure anonymity.

### Statistical analysis

IBM SPSS Statistics for Windows version 28 (IBM Corp. in Armonk, NY) was used to perform all analyses. As all variables were categorical, descriptive statistics including frequency and cross-tabulation with percentages were used to describe sample characteristics. Due to the dichotomous nature of the outcome variable, logistic regression was used to estimate the association between emotional abuse in childhood and somatic and mental specialist healthcare utilization in adulthood. The results are presented as odds ratios (ORs) and corresponding confidence intervals (CIs), and the estimates were considered statistically significant if they met *p* < .05 level. We examined possible interactions between ethnicity and somatic and mental specialist healthcare utilization in adulthood by adding an interaction term between ethnicity and childhood abuse in the regression model, and conducted stratified analyses where applicable. If no interaction was evident, we included ethnicity as a confounder in the model.

### Ethics

The questionnaire used in SAMINOR 2 has previously been approved by The Regional Committee of Research Ethics (REC) (ID 2011/1840) and the Norwegian Centre for Research Data (NSD). All participants gave informed consent to participate when returning the questionnaire. The present analysis was submitted for approval to both the REC (ID 374,706) and the NSD (ID 331,086), both of which concluded that approval was not required. The present study also obtained approval from the Ethics expert committee for Sami Health Research (ID 13/21) - an expert committee selected by the Sami Parliament that must consider any research on Sami health.

## Results

### Participant characteristics

Analyses of the association between emotional abuse in childhood and somatic specialist healthcare utilization in adulthood included 8681 participants: 19% (*n* = 1689) Sami and 81% (*n* = 6692) non-Sami. Analyses of the association between emotional abuse in childhood and mental specialist healthcare utilization in adulthood included 8459 participants: 20% (*n* = 1658) Sami and 80% (*n* = 6801) non-Sami.

Among all (*n* = 8681), 58% reported somatic specialist healthcare utilization in the last 12 months. Among all (*n* = 8459), 3.3% reported mental specialist healthcare utilization in the last 12 months. See Table 1 for background characteristics of the participants. In the whole sample, 13% of the participants reported emotional abuse in childhood. In Table 2, the number of participants reporting on emotional abuse in childhood and healthcare use is outlined, by ethnicity.

**Table 1:** Participant characteristics by ethnicity. The SAMINOR 2 questionnaire survey

Variables		Reporting on somatic healthcare utilization (*n* = 8681)		Reporting on mental healthcare utilization (*n* = 8459)	
		Sami *N* = 1689 (19%)	Non-Sami *N* = 6692 (81%)	Sami *N* = 1658 (20%)	Non-Sami *N* = 6801 (80%)
Gender	Female	901 (54%)	3710 (53%)	891 (54%)	3608 (53%)
	Male	788 (47%)	3282 (47%)	767 (46%)	3193 (47%)
Age	18–39	524 (31%)	1851 (26%)	521 (32%)	1820 (27%)
	40–49	347 (21%)	1599 (23%)	338 (20%)	1559 (23%)
	50–69	818 (48%)	3542 (51%)	799 (48%)	3422 (50%)
Educational level, years	0–9	231 (14%)	1001 (14%)		
	10–12	418 (25%)	1990 (29%)		
	13–15	416 (24%)	1911 (27%)		
	≥ 16	624(37%)	2090 (30%)		
	0–12			637 (38%)	2891 (43%)
	≥ 13			1021 (62%)	3910 (57%)

**Table 2:** N (%) of participants reporting exposure to emotional abuse in childhood and the use of somatic and mental healthcare the past 12 months, by ethnicity

		Somatic healthcare utilization				Mental healthcare utilization			
		Sami		Non-Sami		Sami		Non-Sami	
		No (*N* = 982)	Yes (*N* = 707)	No (*N* = 4032)	Yes (*N* = 2960)	No (*N* = 1600)	Yes (*N* = 58)	No (*N* = 6581)	Yes (*N* = 220)
Exposure to emotional abuse in childhood	No	799 (81%)	553 (78%)	3620 (90%)	2593 (88%)	1292 (81%)	37 (64%)	5893 (90%)	135 (61%)
	Yes	183 (19%)	154 (22%)	412 (10%)	367 (12%)	308 (19%)	21 (36%)	688 (10%)	85 (39%)

### The association between emotional abuse in childhood and somatic and mental specialist healthcare utilization in adulthood

Emotional abuse in childhood was significantly associated with somatic specialist healthcare utilization in adulthood (crude OR 1.23, 95% CI 1.08–1.40). The significant association remained after partially adjusting the model for age and after fully adjusting the model for age, gender, ethnicity, and educational level (Table 3).

Emotional abuse in childhood was also significantly associated with the mental specialist healthcare utilization in adulthood (crude OR 4.45, 95% CI 3.46–5.71). The significant association remained after partially adjusting the model for age and after fully adjusting for age, gender, and educational level (Table 3).

**Table 3:** Association between emotional abuse in childhood and somatic and mental specialist healthcare utilization in adulthood. The SAMINOR 2 Questionnaire Survey

		Somatic specialist healthcare utilization				Mental specialist healthcare utilization			
		Age adjusted model		Fully adjusted model*		Age adjusted model		Fully adjusted model**	
		OR	95% CI	OR	95% CI	OR	95% CI	OR	95% CI
Emotional abuse in childhood	No	-		-		-		-	
	Yes	1.29	1.14–1.46	1.31	1.15–1.49	3.98	3.09–5.13	3.99	3.09–5.14

OR: odds ratio, CI: confidence interval *Confounders included in somatic healthcare use: Age, gender, education, & ethnicity. **Confounders included in mental healthcare use: Age, gender, & education.

### Interaction between mental specialist healthcare utilization in adulthood and ethnicity

There was a significant interaction between mental specialist healthcare utilization in adulthood and ethnicity. Therefore, we repeated the analysis and stratified by ethnicity. As few Sami participants reported using mental specialist healthcare in the stratified analyses (*N* = 21), only a univariate analysis was conducted. Participants who reported Sami ethnicity had a lower OR for mental specialist healthcare utilization (crude OR 2.38, 95% CI 1.37–4.13) compared to participants reported non-Sami ethnicity (crude OR 5.40, 95% CI 4.07–7.15). We found no significant interaction of ethnicity on somatic healthcare utilization, and therefore included ethnicity as a possible confounder in this model.

## Discussion

### Main findings

This study aimed to investigate the association between emotional abuse in childhood and somatic and mental specialist healthcare utilization in adulthood in Sami and non-Sami populations. The results demonstrate that emotional abuse in childhood is significantly associated with increased somatic and mental specialist healthcare utilization in adulthood. These significant associations were present also after adjusting for age, gender and educational level. For those who reported emotional abuse in childhood, the odds for reporting mental specialist heathcare were almost four-fold compared to the odds of reporting somatic specialist healthcare. Moreover, the association between emotional abuse in childhood and mental specialist healthcare utilization was weaker among Sami (OR 2.38) than non-Sami participants (OR 5.40). To the best of our knowledge, this is the first population-based study in either a global or indigenous context to investigate the specific impact of emotional abuse in childhood on somatic and mental specialist healthcare utilization in adulthood.

### Association between emotional abuse and healthcare utilization

Our findings are consistent with research that investigated how additional childhood stress affects healthcare utilization in adulthood [32], as well as studies that explored the distinct impacts of sexual and physical childhood abuse on healthcare utilization in adulthood (visits to general practitioners, emergency departments, and hospital admission). A retrospective cohort study [32] investigated how additional childhood stress, including exposure to abuse (sexual, physical, and emotional) and neglect, and growing up in environments affected by issues such domestic abuse, substance misuse, or mental illness, impacts healthcare utilization in adulthood (visits to general practitioners, emergency department visits, and hospital overnight stays). The OR for emergency department visits (OR 1.34) and overnight hospital stays (OR 1.32) are consistent with our study’s findings regarding the association between emotional abuse in childhood and somatic specialist healthcare utilization (OR 1.30). A population-based study examining how sexual and physical abuse in childhood could affect healthcare utilization in adulthood including both primary and secondary healthcare, found significant associations between sexual and physical abuse and emergency department visits, with an OR of 1.84 and 1.74, respectively [33]. Considering that emotional violence is the most frequently reported type of childhood abuse [5, 9], it is noteworthy that our findings are similar to those from other studies that have looked at other types of childhood violence and use of healthcare services. While these studies measured healthcare use in various ways and sometimes included visits to specialists without separating mental from somatic healthcare, our study indicates that making this separation is important due to the distinct difference in associations between somatic and mental specialist healthcare.

### Emotional abuse in childhood and mental specialist healthcare utilization vs. emotional abuse in childhood and somatic specialist healthcare utilization

Our results showed a stronger association between emotional abuse in childhood and mental specialist healthcare utilization compared to somatic specialist healthcare utilization in adulthood. This finding is not unexpected, as a systematic review showed similar results when examining the association with health outcomes [8], with stronger associations between emotional abuse in childhood and psychological disorders compared to somatic diseases. Additionally, emotional abuse in childhood has consistently been linked to various psychological symptoms and disorders, including depression, anxiety, and post-traumatic stress disorder [8, 11, 34–36]. The relationship between emotional violence in childhood and somatic health outcomes is also possibly linked to its impact on neurophysiological development, affecting pathways through neurological, hormonal and chronic inflammation systems that are especially vulnerable during critical sensitive periods of childhood [37–39]. Although there are fewer studies investigating the relationship between childhood abuse and somatic illnesses, some studies have found that adverse experiences in childhood may be a risk factor for somatic diseases in adulthood, such as cardiovascular disease and diabetes mellitus [40, 41]. Taken together, childhood emotional abuse appears to have stronger impact on mental specialist health care utilization than somatic healthcare utilization. However, it is important to consider that only a small number of participants who reported emotional abuse in childhood also reported mental specialist healthcare utilization (3.3% of participants). Therefore, our findings should be considered preliminary.

### Similarities and differences between Sami and non-sami populations

The association between exposure to emotional abuse in childhood and somatic specialist healthcare utilization was the same in both ethnic groups. Compared to other indigenous populations, who tend to have less primary and specialist healthcare utilization and higher hospital admission rates [23], studies have indicated that both the youth and the adult population among Sami and non-Sami in Norway have comparable healthcare utilization [24, 25, 42]. Moreover, an additional study indicated that expenditure on somatic and mental specialist healthcare is comparable in both Sami-majority and Sami-minority municipalities in the same geographical area where the SAMINOR 2 study was conducted [24]. An argument could be made that this is a result of similarities in socioeconomic status between Sami and non-Sami populations in Norway compared to other indigenous populations that tend to have lower socioeconomic status than the majority population in their country, which in turn may restrict their access to healthcare [43]. Additionally, it is possible that the combination of geographical factors, with Sami and non-Sami populations residing in the same geographical regions of Norway, and Norway’s universal healthcare system, may ensure equitable access to healthcare services [44, 45].

Despite equal access and few differences in socioeconomic status among Sami, we found a weaker association between emotional abuse in childhood and mental specialist healthcare utilization among Sami than non-Sami participants. It might be that the Sami face specific challenges in accessing mental healthcare, including cultural and language barriers, limited knowledge about Sami culture among mental healthcare workers, and potential deficiencies in addressing their unique needs [46]. Moreover, the Sami population has been subject to forced assimilations, prejudice, and discrimination from the majority population. As a result, Sami people might lack trust in mental health professionals and be more hesitant to seek help for their mental health issues than non-Sami people. Another possible explanation may be a stronger stigma associated with mental health problems among Sami than non-Sami [47]. The phenomenon of stigma surrounding mental health disorders is prevalent across different societies and can act as a barrier to seeking appropriate help [48, 49]. In conclusion, this absence of cultural and language competence among mental healthcare professionals, the stigma of mental health problems, and the historical trauma of the Sami population, might weaken the association between emotional abuse in childhood and mental specialist healthcare utilization in adulthood among Sami compared to non-Sami populations. Finally, the weaker association between emotional abuse in childhood and mental specialist healthcare utilization in adulthood in Sami participants might be influenced by few participants and lack of statistical power. Analyses stratified by ethnicity resulted in small numbers across the different groups, and we were unable to control for potential confounding variables. Hence, it is crucial to interpret our findings with caution.

In our population, 13% of participants reported being exposed to emotional abuse in childhood. We did not examine the prevalence among Sami and non-Sami specifically. However, Table 2 indicates that more Sami than non-Sami participants report emotional abuse in childhood, which corresponds to other research on indigenous populations [16–19]. There might be several possible explanations for this trend, but harsh assimilation policies, colonization and discrimination appear to be the main hypotheses [21, 50]. However, a discussion on prevalence and of possible causes for any ethnic difference in prevalence is deemed beyond the scope of this paper.

### Strengths and limitations

The large sample size is one of the strengths of this study, as it provides a unique opportunity to examine the impact of emotional abuse in childhood on somatic and mental specialist healthcare utilization in adulthood separately. In addition, SAMINOR 2 data were collected in municipalities with Sami and non-Sami populations [27], enabling the examination of ethnic differences within the same regions. However, the low response rate (27%) may pose a threat to generalizability and introduce non-response bias, and unfortunately, we do not have any information on non-participants. Research has indicated that non-participants in population-based studies tend to be younger and have lower levels of education [51], which may have affected our results. Although we do not have any data on non-participants, the proportion of healthcare utilization we observed corresponds to Norway’s national report on health services, suggesting that the healthcare utilization in our study is representative of the Norwegian population [52].

One major limitation is that emotional violence was defined by one question only. Several questions might have shown a broader picture on this issue and hence given a different result. The use of questions from the NorAQ, a validated questionnaire to measure childhood abuse, is considered a strength of this study [27, 29, 30]. However, the NorAQ questions have not yet been validated in Sami and non-Sami populations in Norway. Due to cultural and linguistic differences, misinterpretations of NorAQ questions cannot be ruled out. Therefore, the results need to be interpreted with caution.

Recall bias might have interfered the results as respondents who struggle with mental and physical health might recall adverse events like childhood abuse in a more negative way [53], and hence amplify the association between childhood abuse and adult health care utilization. On the other hand, studies suggest that it is more common to under-report childhood abuse in adulthood [54], hence weakening the association between childhood abuse and adult health care utilization. Both types of bias might have disrupted the results. Another source of bias is the large age-span between the respondents (18–69 years). Hence the memories and the interpretation of memories might differ throughout the lifespan.

There is no general agreement in the literature on the use of the term “violence” or “abuse”. The term “abuse” is often linked to non-physical behaviours perpetrated by parents/caregivers. In this study the term “abuse” is used, however, it should be noted that we do not have information on perpetrators in this study.

Lastly, the cross-sectional nature of this study does not allow us to determine any causal link between emotional abuse in childhood and specialist healthcare utilization in adulthood. However, as emotional abuse in childhood occurred prior to the reported healthcare utilization in adulthood, we can speculate on the direction of the association.

## Conclusion

In conclusion, our study indicates that emotional abuse experienced in childhood could impact both mental and somatic specialist healthcare utilization in adulthood. This association was stronger for mental specialist healthcare utilization than somatic specialist healthcare utilization. The association between emotional abuse in childhood and mental specialist healthcare utilization was less pronounced among Sami than non-Sami participants, although these results should be interpreted as preliminary due to the few Sami participants reporting emotional abuse in childhood and mental specialist healthcare use. Further research is needed to better understand the reasons for these associations as well as the ethnic differences.

## Data Availability

The data that support the findings of this study were used under license for the current study and are therefore not publicly available. Data are available from the SAMINOR Study upon reasonable request (www.saminor.no), but restrictions apply to the availability of these data, due to Norwegian privacy regulations.

## Abbreviations

CI: Confidence interval
NorAQ: NorVold Abuse Questionnaire
NSD: Norwegian Centre for Research Data
OR: Odds Ratio
REC: The Regional Committee of Research Ethics
SAMINOR 2: SAMINOR 2 Questionnaire survey
SSB: Statistics Norway
